# Crystal structure of bis­(1-hexyl-*N*,*N*-di­methyl­pyridinium) bis­(maleo­nitrile­dithiol­ato)nickelate(II)

**DOI:** 10.1107/S2056989016011191

**Published:** 2016-07-26

**Authors:** Shan-Shan Yu, Hui Zhang

**Affiliations:** a Department of Environmental Science, Nanjing Xiaozhuang College, Nanjing 211171, People’s Republic of China; b Huabao Food Flavor & Fragrance (Shanghai) Co., Ltd, Shanghai, 201821, People’s Republic of China

**Keywords:** crystal structure, maleo­nitrile­dithio­late, hydro­carbon chain, synthesis

## Abstract

The asymmetric unit of the title compound, (C_13_H_23_N_2_)_2_[Ni(C_4_N_2_S_2_)_2_], consists of a 1-hexyl-*N*,*N*-di­methyl­pyridinium cation and one half of a [Ni(mnt)_2_]^2−^ dianion (mnt^2−^ = maleo­nitrile­dithiol­ate) in which the Ni^2+^ cation lies on a crystallographic inversion centre. The square-planar coordination about Ni^2+^ comprises four S atoms from two bidentate chelate mnt^2−^ ligands [Ni—S = 2.1791 (9) and 2.1810 (8) Å, and S—Ni—S bite angle = 91.93 (3)°]. The hydro­carbon chains of cations show *trans*-planar conformations and lie approximately parallel to the long mol­ecular axis of the [Ni(mnt)_2_]^2−^ anions, giving stacks along the *c* axis. The anions and cations form layers lying parallel to the *bc* plane. Only weak C—H⋯Ni and C—H⋯π associations are present in the crystal packing.

## Abstract 2

In the title salt, the1-hexyl-*N*,*N*-di­methyl­pyridinium cations possess an extended chain conformation, while the bis­(maleo­nitrile­dithiol­ato)nicklate(II) complex anion has a square-planar NiS_4_ geometry comprising four S-donor atoms from two bidentate chelate comprising maleo­nitrile­dithiol­ate ligands, with the Ni^2+^ cation lying on a crystallographic mirror plane. The crystal has a layered structure consisting of alternating cations and anions.

## Chemical context   

Mol­ecular solids based on transition metal di­thiol­ene complexes have attracted much inter­est in recent years, not only regarding fundamental research of magnetic inter­actions and magneto-structural correlations but also in the development of new functional-mol­ecule-based materials (Robertson & Cronin, 2002 ▸). Much work has been performed in mol­ecular solids based on *M*[di­thiol­ene]_2_ complexes because of their application as building blocks in mol­ecular-based materials showing magnetic, superconducting and optical properties (Nishijo *et al.*, 2000 ▸; Ni *et al.*, 2004 ▸, Ren *et al.*, 2004 ▸). In our previous studies, we have investigated the effect of the introduction of mobile organic cations into the rigid [Ni(mnt)_2_]^2−^ spin system and created some multi-functional compounds (Yu *et al.*, 2012 ▸, 2013 ▸; Duan *et al.*, 2011 ▸). In order to further explore the correlation between the structural features of the counter-cations and the stacking patterns of the anions as well as their physical properties, we have designed and synthesized the soft 1-hexyl-*N*,*N*-di­methyl­pyridinium cation and combined it with the [Ni(mnt)_2_]^2−^ dianion, giving the title compound, (C_13_H_23_N_2_)_2_[Ni(C_4_N_2_S_2_)_2_], (I), and the crystal structure is reported herein.

## Structural comment   

**Figure 1:**
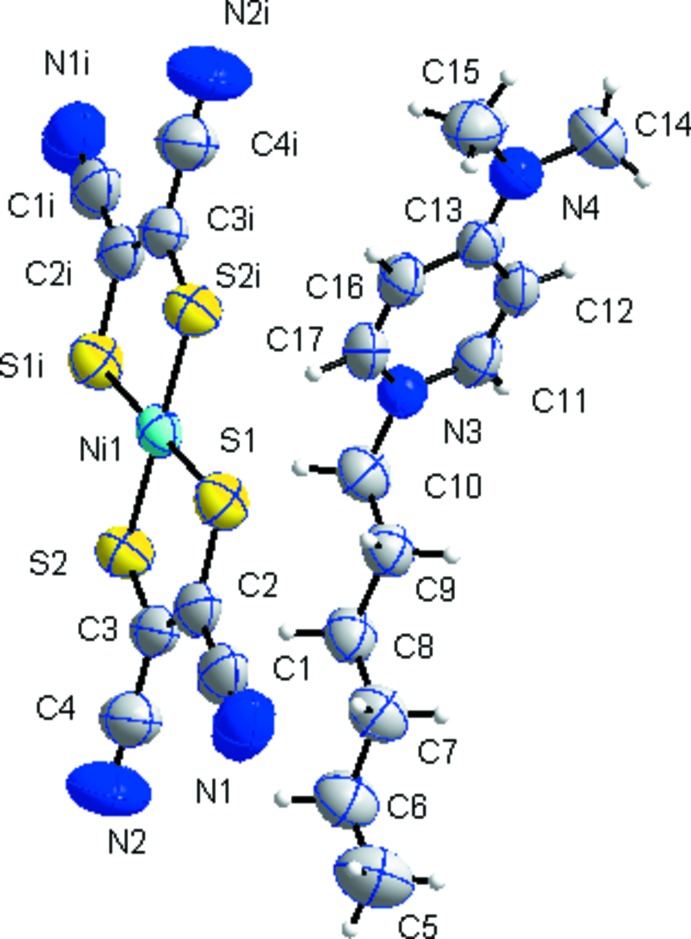
The atom-numbering scheme in the mol­ecular structure of (I), showing the cation and the centrosymmetric dianion, with displacement ellipsoids drawn at the 30% probability level. [Symmetry code: (i) −*x* + 1, −*y*, −*z*.]

In the structure of (I) (Fig. 1 ▸), the asymmetric unit comprises a 1-hexyl-*N*,*N*-di­methyl­pyridinium cation and one half of an [Ni(mnt)_2_]^2−^ dianion (mnt^2−^ = maleo­nitrile­dithiol­ate). The Ni^2+^ ion lies on a crystallographic inversion centre (Fig. 1 ▸). The complex dianion possesses an approximately planar geometry with Ni—S bond lengths of the bidentate ligands of 2.1791 (9) and 2.1810 (8) Å and an1 S—Ni—S2 bite angle of 91.93 (3)°. These values are in good agreement with those found in various [Ni(mnt)_2_]^2−^ compounds (Duan *et al.*, 2014*a* ▸).
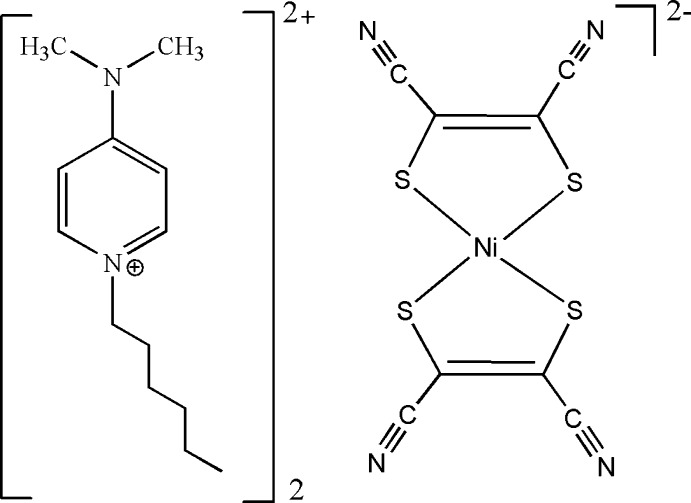


**Figure 2:**
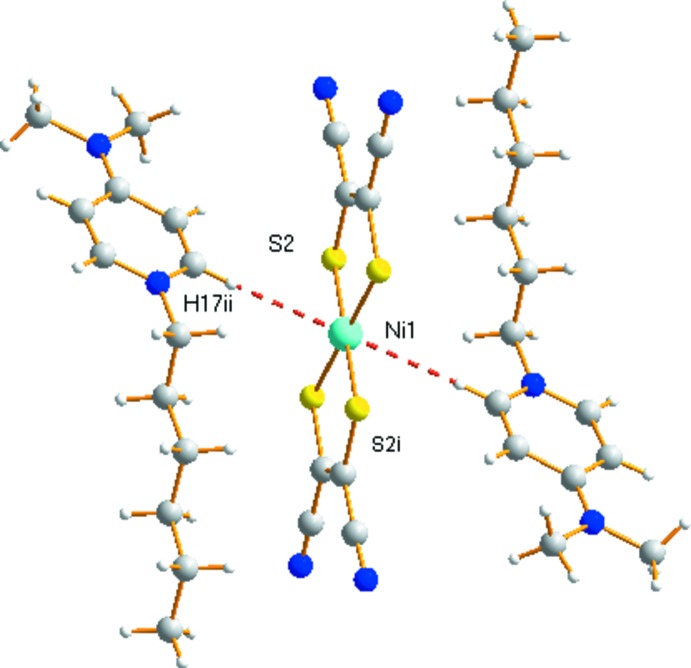
The Ni⋯H—C contact between the layered cation and anion species in (I). [Symmetry code: (ii) −*x* + 1, −*y* + 1, −*z*.]

The hydro­carbon chain of the cation is slightly disrupted close to the pyridyl ring in the completely *trans*-planar conformation, with a chain to pyridyl ring dihedral angle of 83.03 (19)°. The direction of the hydro­carbon chains is approximately parallel to the long mol­ecular axis of the anions, with a dihedral angle between the mol­ecular plane of the hydro­carbon chain and that of the anion (defined by S1,S2,S2^i^,S1^i^) of 10.76 (18)° [symmetry code: (i) −*x* + 1, −*y*, −*z*]. Between the cation and anion there is a novel Ni1⋯H—C17^ii^ inter­action (H⋯Ni = 2.72 Å) (Fig. 2 ▸) [symmetry code: (ii) −*x* + 1, −*y* + 1, −*z*].

## Supra­molecular features   

**Figure 4:**
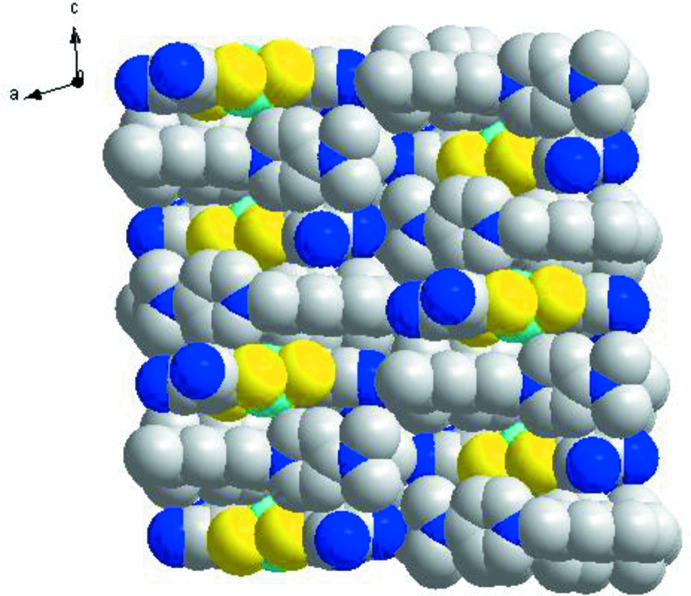
The packing diagram, viewed along *b*, of the crystals of (I).

In the crystal of (I), both the anions and cations form layers lying parallel to the *bc* plane (Figs. 2 ▸ and 3 ▸). In the anion layer, two neighboring [Ni(mnt)_2_]^2−^ anions are associated *via* side-to-side stacking with typical inter­atomic separations of 8.713 (1) Å (Ni1⋯Ni1^ii^), and 6.218 (3) Å (S1⋯S2^ii^). The cations are arranged into bilayers, also lying parallel to the *ab* plane. In each layer, the cations exhibit an anti­parallel arrangement. The cation and anion layers stack alternately, forming columns which extend along *c* (Fig. 4 ▸).

In the crystal there are no formal hydrogen-bonding inter­actions. However, there are two weak C17—H⋯π associations to the chelate ring of the [Ni(mnt)_2_]^2−^ dianions (*Cg*1, defined by Ni1,S1,C2,C3,S2): to *Cg*1^ii^ and *Cg*1^iii^ (H⋯*Cg* = 2.77 Å) [symmetry code: (iii) *x*, *y* − 1, *z*].

## Database survey   

In the structures of [Ni(mnt)_2_]^2−^ complex dianions, chair-shaped organic compounds have been chosen as counter-cations and a series of compounds with segregated anion and cation stacks have been obtained (Pei *et al.*, 2012 ▸; Tian *et al.*, 2009 ▸; Ren *et al.*, 2006 ▸). In addition, with [Ni(mnt)_2_]^2−^ anions, nine compounds with 1-alkyl-4-amino­pyridinium analogs as counter-cations have been synthesized (Duan *et al.*, 2014*b* ▸). In these, the hydro­carbon chains of the counter-ions adopt *trans*-planar conformations and mixed stacking structures of anions and cations are also observed

## Synthesis and crystallization   

Disodium maleo­nitrile­dithiol­ate (2.0 mmol) and nickel(II) chloride hexa­hydrate (1.0 mmol) were mixed with stirring in water (20 mL) at room temperature. Subsequently, a solution of 1-hexyl-*N*,*N*-di­methyl­pyridinium iodide (1.0 mmol) in methanol (10 mL) was added to the mixture and the red precipitate that was immediately formed was filtered off and washed with methanol. The crude product was recrystallized in acetone (20 mL) to give red block-shaped crystals which were used in the X-ray analysis.

## Refinement   

Crystal data, data collection and refinement details are summarized in Table 1 ▸. The H atoms were placed in geometrically idealized positions (C—H = 0.93–0.98 Å) and refined as riding with *U*_iso_(H) = 1.2*U*_eq_(aromatic or methyl­ene) or 1.5*U*_eq_(meth­yl).

## Supplementary Material

Crystal structure: contains datablock(s) global, I. DOI: 10.1107/S2056989016011191/zs2364sup1.cif

Structure factors: contains datablock(s) I. DOI: 10.1107/S2056989016011191/zs2364Isup2.hkl

CCDC reference: 1491765

Additional supporting information: crystallographic information; 3D view; checkCIF report

## Figures and Tables

**Figure 3:**
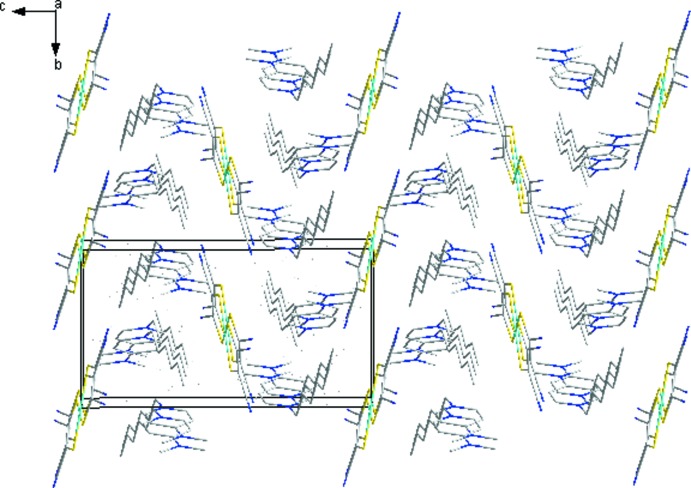
Alternating anion and cation layers, which lie parallel to the crystallographic *ab* plane.

**Table 1:** Experimental details

Crystal data	
Chemical formula	(C_13_H_23_N_2_)_2_[Ni(C_4_N_2_S_2_)_2_]
*M*_r_	753.72
Crystal system, space group	Monoclinic, *P*2_1_/*c*
Temperature (K)	296
*a*, *b*, *c* (Å)	14.241 (2), 8.7129 (14), 16.393 (3)
β (°)	102.560 (2)
*V* (Å^3^)	1985.4 (6)
*Z*	2
Radiation type	Mo *K*α
μ (mm^−1^)	0.73
Crystal size (mm)	0.40 × 0.20 × 0.20

Data collection	
Diffractometer	Bruker SMART CCD area-detector
Absorption correction	Multi-scan (*SADABS*; Bruker, 2000 ▸)
*T*_min_, *T*_max_	0.811, 0.903
No. of measured, independent and observed [*I* > 2σ(*I*)] reflections	16992, 4553, 2772
*R*_int_	0.065
(sin θ/λ)_max_ (Å^−1^)	0.651

Refinement	
*R*[*F* ^2^ > 2σ(*F* ^2^)], *wR*(*F* ^2^), *S*	0.047, 0.142, 1.00
No. of reflections	4553
No. of parameters	217
H-atom treatment	H-atom parameters constrained
Δρ_max_, Δρ_min_ (e Å^−3^)	0.28, −0.47

Computer programs: *SMART* and *SAINT* (Bruker, 2000 ▸), *SHELXS97*, *SHELXL97* and *SHELXTL* (Sheldrick, 2008 ▸).

## supplementary crystallographic information

### Crystal data

**Table:**

(C_13_H_23_N_2_)_2_[Ni(C_4_N_2_S_2_)_2_]	*F*(000) = 796
*M**_r_* = 753.72	*D*_x_ = 1.261 Mg m^−^^3^
Monoclinic, *P*2_1_/*c*	Mo *K*α radiation, λ = 0.71073 Å
Hall symbol: -P 2ybc	Cell parameters from 773 reflections
*a* = 14.241 (2) Å	θ = 2.6–21.2°
*b* = 8.7129 (14) Å	µ = 0.73 mm^−^^1^
*c* = 16.393 (3) Å	*T* = 296 K
β = 102.560 (2)°	Prism, red
*V* = 1985.4 (6) Å^3^	0.40 × 0.20 × 0.20 mm
*Z* = 2	

### Data collection

**Table:**

Bruker SMART CCD area-detector diffractometer	4553 independent reflections
Radiation source: fine-focus sealed tube	2772 reflections with *I* > 2σ(*I*)
Graphite monochromator	*R*_int_ = 0.065
phi and ω scans	θ_max_ = 27.5°, θ_min_ = 1.5°
Absorption correction: multi-scan (SADABS; Bruker, 2000)	*h* = −18→17
*T*_min_ = 0.811, *T*_max_ = 0.903	*k* = −11→11
16992 measured reflections	*l* = −21→20

### Refinement

**Table:**

Refinement on *F*^2^	Primary atom site location: structure-invariant direct methods
Least-squares matrix: full	Secondary atom site location: difference Fourier map
*R*[*F*^2^ > 2σ(*F*^2^)] = 0.047	Hydrogen site location: inferred from neighbouring sites
*wR*(*F*^2^) = 0.142	H-atom parameters constrained
*S* = 1.00	*w* = 1/[σ^2^(*F*_o_^2^) + (0.0734*P*)^2^] where *P* = (*F*_o_^2^ + 2*F*_c_^2^)/3
4553 reflections	(Δ/σ)_max_ < 0.001
217 parameters	Δρ_max_ = 0.28 e Å^−^^3^
0 restraints	Δρ_min_ = −0.47 e Å^−^^3^

### Special details

**Table:**

Geometry. All esds (except the esd in the dihedral angle between two l.s. planes) are estimated using the full covariance matrix. The cell esds are taken into account individually in the estimation of esds in distances, angles and torsion angles; correlations between esds in cell parameters are only used when they are defined by crystal symmetry. An approximate (isotropic) treatment of cell esds is used for estimating esds involving l.s. planes.
Refinement. Refinement of F^2^ against ALL reflections. The weighted R-factor wR and goodness of fit S are based on F^2^, conventional R-factors R are based on F, with F set to zero for negative F^2^. The threshold expression of F^2^ > 2sigma(F^2^) is used only for calculating R-factors(gt) etc. and is not relevant to the choice of reflections for refinement. R-factors based on F^2^ are statistically about twice as large as those based on F, and R- factors based on ALL data will be even larger.

### Fractional atomic coordinates and isotropic or equivalent isotropic displacement parameters (Å^2^)

**Table:**

	*x*	*y*	*z*	*U*_iso_*/*U*_eq_	
Ni1	0.5000	0.0000	0.0000	0.05898 (18)	
S1	0.54081 (6)	0.24019 (9)	0.02097 (5)	0.0763 (2)	
S2	0.64932 (5)	−0.07007 (9)	0.01137 (5)	0.0720 (2)	
N1	0.7570 (3)	0.4849 (4)	0.0913 (2)	0.1208 (12)	
N2	0.8973 (2)	0.0748 (5)	0.0871 (2)	0.1362 (13)	
N3	0.55955 (17)	0.9719 (2)	0.27888 (15)	0.0658 (6)	
N4	0.83752 (17)	0.8050 (3)	0.34593 (14)	0.0743 (6)	
C1	0.7170 (3)	0.3720 (4)	0.07145 (18)	0.0856 (9)	
C2	0.6657 (2)	0.2325 (3)	0.04658 (16)	0.0705 (7)	
C3	0.7121 (2)	0.0976 (4)	0.04262 (16)	0.0689 (7)	
C4	0.8156 (3)	0.0868 (4)	0.0665 (2)	0.0921 (10)	
C5	0.0487 (3)	0.6987 (5)	0.1394 (3)	0.1196 (13)	
H5A	0.0644	0.6439	0.0933	0.179*	
H5B	−0.0169	0.7329	0.1244	0.179*	
H5C	0.0568	0.6322	0.1871	0.179*	
C6	0.1144 (2)	0.8359 (4)	0.1604 (2)	0.0938 (10)	
H6A	0.1027	0.9054	0.1130	0.113*	
H6B	0.0982	0.8898	0.2072	0.113*	
C7	0.2197 (2)	0.7958 (4)	0.1820 (2)	0.0845 (9)	
H7A	0.2355	0.7409	0.1353	0.101*	
H7B	0.2312	0.7268	0.2296	0.101*	
C8	0.2867 (2)	0.9327 (3)	0.20257 (19)	0.0748 (8)	
H8A	0.2693	0.9910	0.2475	0.090*	
H8B	0.2785	0.9990	0.1540	0.090*	
C9	0.39199 (19)	0.8855 (3)	0.22865 (18)	0.0716 (7)	
H9A	0.3997	0.8152	0.2756	0.086*	
H9B	0.4103	0.8316	0.1827	0.086*	
C10	0.4579 (2)	1.0208 (3)	0.2530 (2)	0.0779 (8)	
H10A	0.4510	1.0907	0.2060	0.093*	
H10B	0.4395	1.0754	0.2987	0.093*	
C11	0.6025 (2)	0.9523 (3)	0.36010 (17)	0.0683 (7)	
H11	0.5689	0.9780	0.4008	0.082*	
C12	0.6928 (2)	0.8963 (3)	0.38405 (16)	0.0677 (7)	
H12	0.7193	0.8825	0.4406	0.081*	
C13	0.7480 (2)	0.8581 (3)	0.32450 (16)	0.0626 (7)	
C14	0.8852 (2)	0.7825 (5)	0.4335 (2)	0.1024 (11)	
H14A	0.8845	0.8770	0.4635	0.154*	
H14B	0.9505	0.7509	0.4370	0.154*	
H14C	0.8519	0.7048	0.4577	0.154*	
C15	0.8934 (2)	0.7713 (4)	0.2824 (2)	0.0950 (10)	
H15A	0.8617	0.6922	0.2457	0.143*	
H15B	0.9567	0.7374	0.3094	0.143*	
H15C	0.8983	0.8624	0.2506	0.143*	
C16	0.7002 (2)	0.8802 (3)	0.23995 (16)	0.0669 (7)	
H16	0.7318	0.8563	0.1975	0.080*	
C17	0.6098 (2)	0.9351 (3)	0.22026 (17)	0.0706 (7)	
H17	0.5806	0.9484	0.1642	0.085*	

### Atomic displacement parameters (Å^2^)

**Table:**

	*U*^11^	*U*^22^	*U*^33^	*U*^12^	*U*^13^	*U*^23^
Ni1	0.0627 (3)	0.0692 (3)	0.0450 (3)	0.0056 (2)	0.0115 (2)	−0.00043 (19)
S1	0.0775 (5)	0.0704 (4)	0.0804 (5)	0.0057 (4)	0.0161 (4)	−0.0003 (4)
S2	0.0666 (5)	0.0811 (5)	0.0697 (4)	0.0073 (4)	0.0176 (3)	−0.0089 (4)
N1	0.154 (3)	0.104 (2)	0.096 (2)	−0.047 (2)	0.011 (2)	−0.0003 (17)
N2	0.068 (2)	0.163 (3)	0.170 (4)	0.001 (2)	0.008 (2)	−0.009 (3)
N3	0.0689 (15)	0.0638 (13)	0.0647 (14)	0.0041 (11)	0.0145 (11)	0.0064 (10)
N4	0.0661 (15)	0.0834 (16)	0.0719 (15)	−0.0009 (12)	0.0114 (12)	0.0088 (12)
C1	0.100 (2)	0.095 (2)	0.0589 (17)	−0.018 (2)	0.0110 (16)	0.0051 (16)
C2	0.0776 (19)	0.0844 (19)	0.0497 (15)	−0.0099 (16)	0.0145 (13)	0.0027 (13)
C3	0.0636 (17)	0.094 (2)	0.0504 (15)	−0.0048 (15)	0.0156 (13)	0.0020 (13)
C4	0.075 (2)	0.107 (3)	0.093 (2)	−0.006 (2)	0.0149 (18)	−0.0019 (19)
C5	0.092 (3)	0.121 (3)	0.138 (3)	−0.001 (2)	0.008 (2)	−0.024 (3)
C6	0.080 (2)	0.093 (2)	0.105 (2)	0.0158 (18)	0.0139 (19)	−0.0008 (19)
C7	0.078 (2)	0.083 (2)	0.090 (2)	0.0143 (17)	0.0131 (16)	−0.0048 (17)
C8	0.0730 (19)	0.0747 (17)	0.0780 (19)	0.0143 (15)	0.0193 (15)	0.0096 (15)
C9	0.0698 (18)	0.0703 (17)	0.0746 (18)	0.0099 (14)	0.0154 (14)	0.0063 (14)
C10	0.073 (2)	0.0718 (18)	0.088 (2)	0.0142 (15)	0.0168 (17)	0.0060 (15)
C11	0.081 (2)	0.0683 (16)	0.0592 (17)	−0.0066 (14)	0.0231 (15)	−0.0001 (13)
C12	0.080 (2)	0.0693 (16)	0.0517 (15)	−0.0069 (14)	0.0110 (13)	0.0076 (12)
C13	0.0690 (18)	0.0560 (14)	0.0618 (16)	−0.0084 (13)	0.0117 (13)	0.0048 (12)
C14	0.083 (2)	0.122 (3)	0.090 (2)	0.008 (2)	−0.0071 (18)	0.019 (2)
C15	0.074 (2)	0.100 (2)	0.113 (3)	0.0033 (18)	0.0271 (19)	0.007 (2)
C16	0.0720 (18)	0.0781 (17)	0.0531 (15)	0.0012 (14)	0.0196 (13)	0.0047 (12)
C17	0.078 (2)	0.0781 (17)	0.0551 (15)	0.0001 (15)	0.0140 (14)	0.0096 (13)

### Geometric parameters (Å, º)

**Table:**

Ni1—S1	2.1791 (9)	C16—C17	1.345 (4)
Ni1—S2	2.1810 (8)	C5—H5A	0.9600
Ni1—S1^i^	2.1791 (9)	C5—H5B	0.9600
Ni1—S2^i^	2.1810 (8)	C5—H5C	0.9600
S1—C2	1.738 (3)	C6—H6A	0.9700
S2—C3	1.731 (3)	C6—H6B	0.9700
N1—C1	1.148 (5)	C7—H7A	0.9700
N2—C4	1.144 (5)	C7—H7B	0.9700
N3—C10	1.480 (4)	C8—H8A	0.9700
N3—C11	1.350 (4)	C8—H8B	0.9700
N3—C17	1.356 (4)	C9—H9A	0.9700
N4—C14	1.462 (4)	C9—H9B	0.9700
N4—C15	1.471 (4)	C10—H10A	0.9700
N4—C13	1.330 (4)	C10—H10B	0.9700
C1—C2	1.431 (5)	C11—H11	0.9300
C2—C3	1.357 (4)	C12—H12	0.9300
C3—C4	1.444 (5)	C14—H14A	0.9600
C5—C6	1.511 (6)	C14—H14B	0.9600
C6—C7	1.505 (4)	C14—H14C	0.9600
C7—C8	1.519 (4)	C15—H15A	0.9600
C8—C9	1.524 (4)	C15—H15B	0.9600
C9—C10	1.506 (4)	C15—H15C	0.9600
C11—C12	1.351 (4)	C16—H16	0.9300
C12—C13	1.420 (4)	C17—H17	0.9300
C13—C16	1.418 (4)		
			
Ni1—S1—C2	103.00 (9)	C7—C6—H6B	109.00
Ni1—S2—C3	102.72 (11)	H6A—C6—H6B	108.00
S1—Ni1—S2	91.93 (3)	C6—C7—H7A	109.00
S1—Ni1—S1^i^	180.00	C6—C7—H7B	109.00
S1—Ni1—S2^i^	88.07 (3)	C8—C7—H7A	109.00
S1^i^—Ni1—S2	88.07 (3)	C8—C7—H7B	109.00
S2—Ni1—S2^i^	180.00	H7A—C7—H7B	108.00
S1^i^—Ni1—S2^i^	91.93 (3)	C7—C8—H8A	109.00
C11—N3—C17	118.3 (2)	C7—C8—H8B	109.00
C10—N3—C11	121.6 (2)	C9—C8—H8A	109.00
C10—N3—C17	120.0 (2)	C9—C8—H8B	109.00
C14—N4—C15	117.5 (2)	H8A—C8—H8B	108.00
C13—N4—C14	121.3 (2)	C8—C9—H9A	109.00
C13—N4—C15	121.2 (2)	C8—C9—H9B	109.00
N1—C1—C2	179.1 (4)	C10—C9—H9A	109.00
C1—C2—C3	121.7 (3)	C10—C9—H9B	109.00
S1—C2—C1	117.9 (2)	H9A—C9—H9B	108.00
S1—C2—C3	120.4 (2)	N3—C10—H10A	109.00
C2—C3—C4	121.5 (3)	N3—C10—H10B	109.00
S2—C3—C2	121.3 (2)	C9—C10—H10A	109.00
S2—C3—C4	117.2 (3)	C9—C10—H10B	109.00
N2—C4—C3	177.9 (4)	H10A—C10—H10B	108.00
C5—C6—C7	114.0 (3)	N3—C11—H11	119.00
C6—C7—C8	114.6 (3)	C12—C11—H11	119.00
C7—C8—C9	112.5 (2)	C11—C12—H12	119.00
C8—C9—C10	112.4 (2)	C13—C12—H12	119.00
N3—C10—C9	111.4 (2)	N4—C14—H14A	109.00
N3—C11—C12	122.1 (3)	N4—C14—H14B	110.00
C11—C12—C13	121.3 (2)	N4—C14—H14C	110.00
N4—C13—C12	122.8 (2)	H14A—C14—H14B	109.00
N4—C13—C16	122.3 (2)	H14A—C14—H14C	109.00
C12—C13—C16	114.9 (3)	H14B—C14—H14C	109.00
C13—C16—C17	120.9 (3)	N4—C15—H15A	109.00
N3—C17—C16	122.6 (3)	N4—C15—H15B	109.00
C6—C5—H5A	110.00	N4—C15—H15C	109.00
C6—C5—H5B	109.00	H15A—C15—H15B	110.00
C6—C5—H5C	109.00	H15A—C15—H15C	109.00
H5A—C5—H5B	109.00	H15B—C15—H15C	109.00
H5A—C5—H5C	109.00	C13—C16—H16	120.00
H5B—C5—H5C	110.00	C17—C16—H16	120.00
C5—C6—H6A	109.00	N3—C17—H17	119.00
C5—C6—H6B	109.00	C16—C17—H17	119.00
C7—C6—H6A	109.00		
			
S2—Ni1—S1—C2	−6.92 (10)	C14—N4—C13—C16	−179.7 (3)
S2^i^—Ni1—S1—C2	173.09 (10)	C15—N4—C13—C12	−178.1 (3)
S1—Ni1—S2—C3	7.10 (10)	S1—C2—C3—S2	0.5 (3)
S1^i^—Ni1—S2—C3	−172.90 (10)	S1—C2—C3—C4	−177.7 (2)
Ni1—S1—C2—C1	−174.9 (2)	C1—C2—C3—S2	−179.3 (2)
Ni1—S1—C2—C3	5.3 (2)	C1—C2—C3—C4	2.5 (4)
Ni1—S2—C3—C2	−6.1 (2)	C5—C6—C7—C8	179.5 (3)
Ni1—S2—C3—C4	172.2 (2)	C6—C7—C8—C9	176.8 (3)
C17—N3—C11—C12	−0.6 (4)	C7—C8—C9—C10	−177.2 (3)
C10—N3—C17—C16	−175.5 (2)	C8—C9—C10—N3	179.4 (2)
C11—N3—C17—C16	0.0 (4)	N3—C11—C12—C13	1.3 (4)
C10—N3—C11—C12	174.8 (2)	C11—C12—C13—N4	178.9 (3)
C11—N3—C10—C9	−96.8 (3)	C11—C12—C13—C16	−1.3 (4)
C17—N3—C10—C9	78.5 (3)	N4—C13—C16—C17	−179.6 (3)
C14—N4—C13—C12	0.0 (4)	C12—C13—C16—C17	0.7 (4)
C15—N4—C13—C16	2.2 (4)	C13—C16—C17—N3	−0.1 (4)

Symmetry code: (i) −*x*+1, −*y*, −*z*.
